# Allyl Isothiocyanate: A TAS2R38 Receptor-Dependent Immune Modulator at the Interface Between Personalized Medicine and Nutrition

**DOI:** 10.3389/fimmu.2021.669005

**Published:** 2021-04-20

**Authors:** Hoai T. T. Tran, Rebecca Stetter, Corinna Herz, Jenny Spöttel, Mareike Krell, Franziska S. Hanschen, Monika Schreiner, Sascha Rohn, Maik Behrens, Evelyn Lamy

**Affiliations:** ^1^ Molecular Preventive Medicine, University Medical Center and Faculty of Medicine—University of Freiburg, Freiburg, Germany; ^2^ Institute of Food Technology and Food Chemistry, Technical University of Berlin, Berlin, Germany; ^3^ Plant Quality and Food Security, Leibniz Institute of Vegetable and Ornamental Crops, Großbeeren, Germany; ^4^ Section II: Metabolic Function, Chemoreception & Biosignals, Leibniz-Institute for Food Systems Biology at the Technical University of Munich, Freising, Germany

**Keywords:** *Brassica* plants, Brassicaceae, isothiocyanates, human bitter taste receptor (TAS2R), TAS2R38, personalized (precision) nutrition, precision medicine, precision health

## Abstract

Understanding individual responses to nutrition and medicine is of growing interest and importance. There is evidence that differences in bitter taste receptor (TAS2R) genes which give rise to two frequent haplotypes, TAS2R38-PAV (functional) and TAS2R38-AVI (non-functional), may impact inter-individual differences in health status. We here analyzed the relevance of the TAS2R38 receptor in the regulation of the human immune response using the TAS2R38 agonist allyl isothiocyanate (AITC) from *Brassica* plants. A differential response in calcium mobilization upon AITC treatment in leucocytes from healthy humans confirmed a relevance of TAS2R38 functionality, independent from cation channel TRPV1 or TRPA1 activation. We further identified a TAS2R38-dependence of MAPK and AKT signaling activity, bactericidal (toxicity against *E. coli*) and anti-inflammatory activity (TNF-alpha inhibition upon cell stimulation). These *in vitro* results were derived at relevant human plasma levels in the low micro molar range as shown here in a human intervention trial with AITC-containing food.

## Introduction

As an important constituent of precision medicine, precision nutrition seeks to develop tailored nutritional approaches to promote and maintain health and to prevent diseases. The strategies consider differential responses to certain individualized food-derived nutrients that arise due to the interaction between nutrients and biological processes. Taste perception is considered as relevant factor for modulating eating behavior and in consequence health and disease risk (1). Bitterness is one key attribute associated with plants from the order Brassicales. This taste is thought to be mediated by interaction of the phytochemical class of glucosinolates (GLS) and their myrosinase-degradation product isothiocyanates (ITC) with the G protein-coupled bitter taste receptor TAS2R38 on the tongue (2). In humans, the bitter taste is perceived by a family of 25 different taste receptors (TAS2Rs) expressed on type II taste receptor cells in the taste bud (3–5). The TAS2R38 was found to respond quite specifically to compounds containing a thiourea [-NH-(C=S)-NH-] or ITC (N=C=S) moiety (6, 7). By today, the TAS2R38 receptor is found to be expressed in many different extragustatory tissues but its physiological relevance in these is still focus for much debate. TAS2R38 protein expression was found recently in peripheral immune cells (neutrophils, monocytes and lymphocytes) (8–10). What makes the receptor especially interesting in the context of precision medicine/nutrition is the occurrence of frequent single nucleotide polymorphisms (SNP) within the human TAS2R38 gene. These SNPs cause amino acid substitutions which result in two common haplotypes, a functional (proline-alanine-valine, PAV) and a non-functional (alanine-valine-isoleucine, AVI) (11). In fact, across almost all human populations worldwide the two alleles occur with high frequencies (12) and, typically, about 25 to 30% of the human population represent non-tasters (13). Sinigrin and its GLS-myrosinase product allyl ITC (AITC) are among the most abundant phytochemicals in *Brassicaceae* (14). The presence of these compounds is reported to contribute to a bitter and pungent taste in *Brassica* vegetables (14, 15) and the taster and non-taster haplotypes of TAS2R38 correlate here well with individuals’ bitter sensitivities for *Brassica* plants (2). As an example, sinigrin was linked with the bitter taste of cooked Brussels sprouts and cauliflower (16, 17). Individuals with a PAV/PAV diplotype have been shown to rate the bitter intensity of *Brassicaceae* vegetables significantly higher than the ones with AVI/AVI (2).

Functional expression studies in HEK293T-Gα16gust44 cells, transiently transfected with the 25 putatively functional ATS2Rs, showed that both sinigrin as well as AITC selectively triggered the taster variant of the TAS2R38 receptor (18). Wu et al. (19) later confirmed their observations using a TAS2R38 receptor-based biosensor. AITC is known for its health promoting potential, including its anti-inflammatory, immune modulatory capacity (20–23). Sinigrin/AITC-containing horseradish root (*Armoracia rusticana*) is used in pharmacological remedies for the treatment of inflammatory diseases including acute sinusitis, bronchitis and urinary tract infection (24, 25). In general, ITC are prominent multi-target compounds interacting with a broad range of signaling pathways and molecules (20). Whether the immune response triggered by AITC is linked to TAS2R38 interaction has not been investigated so far.

Recent findings suggest that a functional TAS2R38 is relevant for the respiratory innate immunity (26) and is proposed as factor for local host defense (27). Further support on this is given by Lee et al. (28). In their study, TAS2R38 has been found to play a role in chronic rhinosinusitis and bacterial detection. Increased susceptibility to chronic rhinosinusitis has also been associated with a non-functional (AVI/AVI) diplotype (28). A non-functional diplotype is linked in cystic fibrosis patients with more severe symptoms (29), it is associated with an increased risk for dental caries (30), increased risk for colorectal (31) and gastric (32) cancer.

In the present study, the effect of AITC on the human innate and adaptive immune system was investigated in dependence on the functional status of the TAS2R38 receptor. Upon AITC treatment, differential effects on calcium mobilization as parameter for G protein-coupled receptor (GPCR) activation were found in human peripheral blood (PBMC) subpopulations and gave first evidence of functional TAS2R38 receptor interaction. Based on these findings, further parameters, with and without stimuli of innate and adaptive immune response were investigated here. A selective bactericidal potential of immune cells from functional TAS2R38 donors was shown using *E. coli* K12 bacteria upon AITC treatment.

## Materials and Methods

### Chemicals

Fetal calf serum (FCS), L-glutamine and phosphate buffered saline (PBS, without Ca and Mg), penicillin-streptomycin (P/S) solution, RPMI-1640 and DMEM without phenol red were from Life Technologies (Darmstadt, Germany). Hank’s Balanced Salt Solution (HBSS) was from PAA Laboratories GmBH (Coelbe, Germany). β-Mercaptoethanol was purchased from Fluka (Buchs, Switzerland). Dimethyl sulfoxide (DMSO; purity > 99%) was purchased from Applichem GmbH (Darmstadt, Germany). Nuclease-free water was from Qiagen (Hilden, Germany). LymphoPrep™ was from Alere Technologies AS (Oslo, Norway). Ionomycin (purity ≥98%), *S*-Nitroso-*N*-Acetyl-D (SNAP), Carboxy-PTIO (cPTIO), capsazepine and A-967079 were from Cayman Europe (Ann Arbor, Michigan, USA), U73122 was purchased from Enzo Life Sciences (Lörrach, Germany). AITC, tween-20, protein standard BSA, ammonium persulfate and lipopolysaccharide (LPS, from *Escherichia coli* O11:B4) were obtained from Sigma-Aldrich (Taufkirchen, Germany). Anti-human CD3 and CD28 functional grade purified antibodies were from eBioscience Affymetrix (Frankfurt, Germany). DAF-FM diacetate, fluo-4-AM and Pluronic™ F-127 were from Thermo Fisher Scientific GmBH (Darmstadt, Germany). DiBAC_4_(3) (Bis-(1,3-dibutylbarbituric acid)trimethine oxonol) was obtained from Biomol (Hamburg, Germany). EDTA (99%, p.a.) was purchased from Serva GmbH (Heidelberg, Germany). Amersham™ ECL Select™ and Hybond ECL Nitrocellulose Membrane was obtained from Ge Healthcare Biosciences AB (Uppsala, Sweden), Quick Start Bradford 1x Dye Reagent was from BioRad Laboratories GmbH (Munich, Germany) and Page Ruler Plus Prestained Protein ladder from Thermo Fisher Scientific (Waltham, Massachusetts, USA). The following primary antibodies were used for immunoblotting: anti-p-ERK1/2 (Thr202/Tyr204, 1:500), anti-ERK1/2 (1:2000, clone L34F12), anti-p-p38 (Thr180/Tyr182, 1:2000), anti-p-Akt (1:2000, Ser 473, clone D9E), anti-Akt (1:1000) from Cell Signalling (Danvers, Massachusetts, USA), and anti-beta-actin (1:10000, clone AC-74) from Sigma-Aldrich Chemie GmbH (Taufkirchen, Germany). The horseradish peroxidase (HRP)-labeled secondary antibodies anti-mouse and anti-rabbit were purchased from Cell Signalling (Danvers, Massachusetts, USA). Formic acid (FA; 98%) and acetonitrile (ACN, ultra LC–MS grade) were purchased from Carl Roth GmbH Co. KG (Karlsruhe, Germany). Trifluoroacetic acid (TFA, 99.9%) was from Carl Roth GmbH Co. KG (Karlsruhe, Germany) and Applichem GmbH (GmbH (Darmstadt, Germany). C18ec solid phase extraction cartridges (3 mL, 200 mg) were gained from Macherey-Nagel GmbH & Co. KG (Düren, Germany). For all aqueous solutions water of Milli-Q quality was used.

### Isolation of Human PBMC

Human PBMC were isolated from fresh peripheral blood of 30 volunteers at the University Medical Center in Freiburg, Germany. Blood was collected from volunteers using Li-heparinized vacutainers (Sarstedt, Nümbrecht, Germany). The volunteers (male and female, aged between 20-35 years) had a normal BMI, were healthy and were non-smokers. PBMC were isolated from blood within 2 h by centrifugation on a LymphoPrep™ gradient (density: 1.077 g/cm^3^) at 1200g for 13 min at room temperature, using 50 mL SepMate™ (Grenoble, France) tubes, were then washed twice with PBS and cell viability and concentration were determined using the trypan blue exclusion test. PBMC were used directly for immunostaining or cultured in RPMI 1640 medium supplemented with 10% heat-inactivated FCS, 2 mM L-glutamine, 100 U/mL penicillin/streptomycin, at 37°C in a humidified incubator with a 5% CO_2_/95% air atmosphere. Cell activation was done by using functional antibodies to CD3 and CD28, or LPS (solvent control: aqua dest).

### Determination of the TAS2R38 Gene Polymorphism

DNA was isolated from 1x10^6^ PBMC using the QIAamp DNA Blood Mini Kit (Qiagen, Hilden, Germany) according to the manufacturer’s protocol. A 831bp fragment of the TAS2R38 gene was amplified using the following oligonucleotides at the indicated final concentration: forward 5′ACCAATGCCTTCGTTTTCTTGGTGA′3 and reverse 5′ CAGCTACCAAGCCATCATCA ′3 (33). PCR reaction was performed in a total volume of 50 µL containing 20 ng DNA, 1 U Taq DNA Polymerase (Life Technologies, Darmstadt, Germany), 0.5 µM forward primer and 0.5 µM reverse primer, dNTP mix at 0.2 mM each (Life Technologies, Darmstadt, Germany) 1.5 mM MgCl and 1x PCR-buffer (Life Technologies, Darmstadt, Germany). The PCR conditions were: 94°C for 3 min followed by 30 cycles of (94°C for 45 sec, 54.2°C for 45 sec and 72°C for 90 sec) and 72°C for 10 min. PCR products were cleaned up using the Monarch PCR & DNA Cleanup Kit (New England Biolabs, Frankfurt am Main, Germany) according to the manufacturer’s protocol. The PCR fragment was sequenced using reverse primer PCR 5′CAGCTACCAAGCCATCATCA′3 to analyze the SNP on position 145 of the gene and the forward primer 5′ GGAAGGCACATGAGGACAAT′3 to analyze the SNPs on position 785 and position 886 by GATC (Konstanz, Germany). The sequences were analyzed using the software Chromas 2.6.4 (Technelysium, South Brisbane, Australia). DNA concentration was analyzed using a NanoDrop (Peq lab, Erlangen, Germany). 5 µl of PCR mix were used to visualize the PCR product in agarose electrophoreses.

### Calcium Flux Assay

PBMC staining for calcium measurement was performed as described before (10). Each sample containing 0.5 x10^6^ cells was analyzed for 800 sec using a FACSCalibur™ (BD Biosciences, Heidelberg, Germany) flow cytometer. After 60 sec, cells were exposed to the test compounds and then measured for another 740 sec. Data were analyzed using FlowJo software (Ashland, Oregon, USA).

### Nitric Oxide (NO) Detection by Flow Cytometry

PBMC (0.25x10^6^) were suspended in DMEM medium and exposed to solvent control, AITC or 100 µM of the NO-donor SNAP (positive control) in 96-well plates for 30-270 min in a humidified incubator (5% CO_2_, 37°C). After that, cells were then loaded with 0.5 µM DAF-FM diacetate prepared in HBSS buffer for another 30 min. DAF-FM loading at this low concentration allows reducing the background fluorescence and improving the signal to noise ratio in a single cell (34). After incubation, the cells were washed twice with PBS, followed by 30 min. de-esterification of the intracellular diacetates in the dark at room temperature. Fluorescence signals (ex 488 nm, em 530± 30 nm) were monitored using a FACSCalibur™ flow cytometer. The median fluorescence intensity (MFI) of each sample was calculated using FlowJo software (Ashland, Oregon, USA). DAF-FM-DA has a detection limit of about 5 nM NO (35).

### Bactericidal Activity

*E. coli* K-12 bacteria were grown in Luria-Bertani medium at 37°C to mid-logarithmic-phase as determined by OD600. Next, PBMC (1x10^6^/mL) were incubated with *E. coli* K-12 at MOI 0.5 in RPMI 1640 medium supplemented with 2% heat-inactivated fetal calf serum and exposed to the test compounds for 120 min in a humidify condition (5% CO_2_, 37°C). Thereafter, 1 µg/mL of the membrane potential dye DiBAC_4_(3) was added and incubated for 10 min at room temperature in the dark. Subsequently, the cells were washed once with PBS and centrifuged at 4500 x g for 10 min. The percentage of depolarized bacteria was determined using a FACSCalibur™ flow cytometer with an excitation wavelength of 493 nm.

### Protein Analysis by Immunoblotting

Proteins of interest were analyzed by immunoblotting as described before (36). Densitometric analysis was done to quantify the immunoblots using Quantity One software (Bio-Rad, Munich, Germany) and normalized against β-actin.

### Quantification of Cytokine Release by Multiplex Bead Technique or ELISA Assay

PBMC (1x10^6^/mL) cultured in RPMI 1640 medium supplemented with 10% heat-inactivated fetal calf serum, 2 mM L-glutamine, 100 U/ml penicillin/streptomycin were stimulated with 0.5 ng/ml CD3/CD28 MAbs and exposed to the test compounds. After 24 h, supernatants were collected and stored at -80°C until analysis using the human MACSplex cytokine 12-kit (Miltenyi Biotec GmbH, Bergisch Gladbach, Germany) according to manufacturer’s protocol to quantify cytokine secretion. Supernatants were also used for photometric quantification by the human TNF-alpha or IL-1β ELISA Ready-Set-Go kit from eBIoscience (Frankfurt, Germany) according to the manufacturer’s instructions.

### Human Food Intervention and Preparation of Blood Serum Samples for Determination of AITC Mercapturic Acid Metabolites

AITC metabolite quantification was carried out in blood plasma samples derived from an intervention study at the University Medical Center in Freiburg, Germany. Samples were from 4 healthy young adults (3 females, 1 male, aged between 30-40 years) having a normal BMI, and were non-smokers. Each subject consumed one time 10 g of a commercially available mustard preparation (Löwensenf extra, Develey Munich, Germany) containing 24.74 ± 1.76 µmol/g fresh weight preformed AITC on an empty stomach with optional consumption of white bread. The intervention was preceded by a 48 h wash-out phase consisting of a glucosinolate/ITC-free diet. After 30 min and optionally 60 min, blood was collected by venipuncture in serum tubes and centrifuged at 2000g for 10 min, 4°C to obtain serum. Subsequently, TFA was added and the samples frozen at -80°C. The preparation of serum samples (300 µL serum and 100 µL TFA) was conducted as described by Kühn et al. (37). The frozen samples were thawed, vortexed (1 min) and centrifuged (4 °C, 10 min, 20.854 x g). The supernatant was transferred to SPE cartridges. Prior to transferring, SPE cartridges were conditioned with acetonitrile (ACN, 3 mL) and equilibrated with water (3 mL). After sample application, the cartridges were washed with formic acid (FA, 3 mL, 0.1% in water) and the metabolites were eluted with FA (3 mL, 0.1% in ACN:water, 90:10, v/v). Subsequently, the eluates were evaporated to dryness with a gentle stream of nitrogen. The samples were re-dissolved in 100 µL FA (0.1% in ACN:water, 90:10, v/v). Sample aliquots of 5 µL were injected into the LC-ESI-MS/MS system.

### LC-ESI-MS/MS Analysis

LC-ESI-MS/MS analysis was performed on an API4000 triple quadrupole mass spectrometer (AB Sciex Germany GmbH, Darmstadt, Germany) equipped with an Agilent 1200 LC system (Agilent Technologies Deutschland GmbH & Co. KG, Waldbronn, Germany). For data acquisition and processing the software Analyst 1.6.1 (AB Sciex Germany GmbH, Darmstadt, Germany) was used. The analysis of the mercapturic acid metabolites (AITC-GSH; AITC-CysGly; AITC-Cys; AITC-NAC) was conducted according to Platz et al. (38) with minor modifications. The separation was performed on a Kinetex C18 column (Phenomenex, 5 μm, 100 Å, 150 x 2.1 mm). The autosampler operated at 4°C and the set temperature for the oven was 20°C. A constant flow of 300 µL/min was used for the mobile phase which consisted of 0.1% FA in water (A) and 0.1% FA in ACN (B). The start composition was 80% of eluent A, being held for 1 min, then eluent B was increased from 20 to 90% within 11 min and held for 4 min. In order to equilibrate the column, A was increased to 90% and held for 5 min. The relevant MS parameters include an entrance potential of -10 V, a desolvation gas temperature of 450°C, ion spray potential of -4.5 kV, gas 1 and gas 2 at 46 psi and a curtain gas pressure at 10 psi. The analysis was done in the negative ionization mode and the quantitation was done by an external calibration in a concentration range between 0.01 μM and 100 μM of each metabolite.

### Statistics

Data were analyzed using GraphPad Prism 6.0 software (La Jolla, California, USA). Results are presented as means +SD. Statistical significance was determined by paired t-test or the ordinary one-way ANOVA followed by Bonferroni correction test. P values < 0.05 (*) were considered statistically significant and < 0.01 (**) were considered highly statistically significant.

### Study Approval

The human intervention trial and blood sampling for *in vitro* experiments were conducted according to the Declaration of Helsinki. The intervention trial was approved by the Ethics Committee of the University of Freiburg with the ethical vote number 54/20. The *in vitro* experiments on human PBMC were approved by the ethics committee of the University of Freiburg with the ethical vote number 597/14. All subjects provided written informed consent before peripheral blood was collected by venipuncture.

## Results

### Functional Activation of the TAS2R38 Receptor by AITC

**Figure 1:**
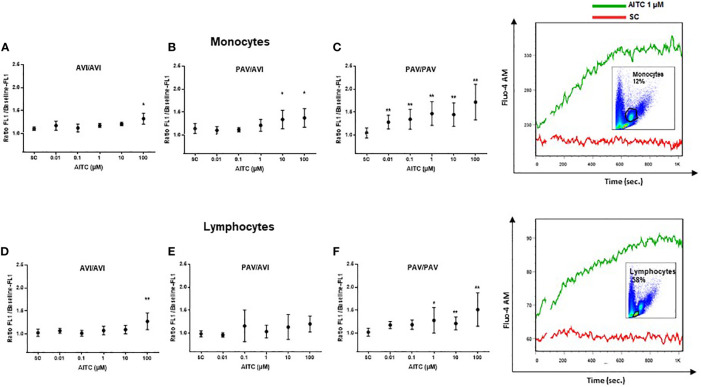
Mobilization of calcium in human PBMC with different TAS2R38 haplotypes after stimulation with AITC. PBMC were isolated and exposed to AITC or solvent control (SC, 0.1% DMSO). Flow cytometry based measurement of calcium flux was detected in monocytes **(A–C)** and lymphocytes **(D–E)** probed with Fluor-4 AM (F_EM:516 nm_) after treatment with compounds. Baseline fluorescence was recorded for 60 sec before exposure. After exposure, fluorescence was measured for another 740 sec. Calcium response was calculated as the ratio of the maximum peak post stimulation to basal level using FlowJo software. Dots represent mean values ± SD, n ≥ 4 of cells from AVI/AVI **(A**, **D),** PAV/AVI **(B**, **E)** and PAV/PAV **(C**, **F)** subjects. Representative pictures of the gating strategy for monocytes or lymphocytes, and calcium flux response to AITC or SC from one subject are given on the right. Significance of difference was determined using paired t-test compared to the respective SC, *p < 0.05, **p < 0.01.

Bitter taste receptors are metabotropic G protein-coupled receptors that can signal through intracellular calcium release involving PLCβ-2 (39). Thus, the potential of AITC to trigger TAS2R38-dependent calcium mobilization in human PBMC was first investigated using flow cytometry. Upon stimulation with AITC, low-level, sustained calcium flux was detected in the monocyte and lymphocyte subpopulation in a genotype-dependent manner while the vehicle alone had no effect (**Figure 1**). A statistically significant calcium flux in monocytes was evident at 0.01 µM for PAV/PAV (22%), 10 µM for PAV/AVI (18%), and 100 µM for AVI/AVI genotypes (20%). In PAV/PAV cells, the calcium influx increased between 0.01 to 1 µM (28% to 35%), then plateaued between 1-10 µM and further increased between 10-100 µM (45% to 72%). Lymphocytes were less sensitive for AITC, but still a genotype-dependent effect on calcium influx could be seen. For AVI/AVI and PAV/AVI, this was evident at 100 µM (24% and 21%, respectively), for PAV/PAV at 1 µM AITC (25%).

**Figure 2:**
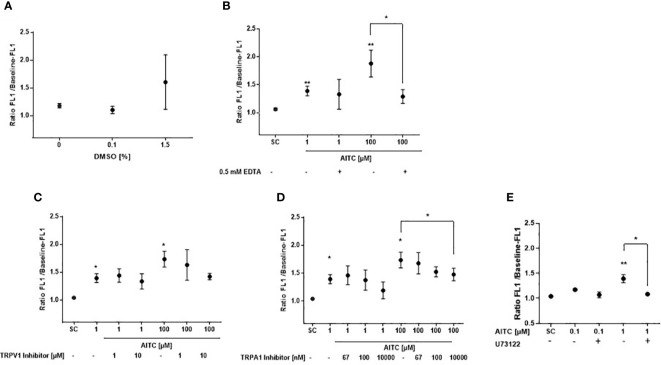
Specificity of T2R38 receptor activation by AITC. PBMC were isolated from PAV/PAV genotype subjects and exposed to **(A)** DMSO or were **(B)** pre-treated with/without 0.5 mM EDTA for 5 min, **(C)** the TRPV1 antagonist capsazepine (CPZ) for 30 min, **(D)** the TRPA1 antagonist A-967079 for 30 min, and **(E)** the PLC-β2 antagonist U73122 for 30 min before AITC exposure. FACS based measurement of calcium flux was then detected in the monocyte subpopulation probed with Fluor-4 AM (F_EM:516 nm_). Baseline fluorescence was recorded for 60 sec. After compound exposure, fluorescence was measured for another 740 sec. Calcium response was calculated as the ratio of the maximum peak post stimulation to basal level using FlowJo software. Dots represent mean values ± SD, n ≥ 3. Significance of difference was determined using the paired t-test compared to the respective solvent control (SC, 0.1% DMSO), *p < 0.05, **p < 0.01.

Morley et al. (40) demonstrated that the solvent DMSO could cause significant calcium release at 1%, and the authors said that already at 0.2% some solvent effects were seen. Moreover, DMSO represents a bona fide, albeit low potency agonist for TAS2R38-PAV with a half maximal (50%) effective concentration (EC_50_) shown in transfected HEK293 cells of 178 mM (~1.3% DMSO solution) and a threshold concentration of approximately >125 mM (~0.9% DMSO solution) (41). In order to exclude that the observed effects of AITC were triggered by the solvent, calcium flux was assessed after cell exposure to DMSO only. At 0.1% DMSO, the highest concentration that the solvent was used in the present study, no effect on calcium release was detected in human (PAV/PAV) monocytes; starting from 1.5% DMSO, a mean calcium increase of 36% compared to untreated cells was observed although statistical significance was, due to considerable fluctuations of the measurements, not reached (**Figure 2A**). We also questioned whether influx from the extracellular environment could be causally involved in the calcium signal detected after AITC treatment. Besides the TAS2R38 receptor, also the transient receptor potential vanilloid 1 and ankyrin 1 (TRPV1 und TRPA1) membrane channels have been reported as a target of AITC. Both, the TRPV1 and TRPA1 are calcium permeable, non-selective cation channels. They function to depolarize the plasma membrane and influx calcium (42). AITC has been shown to activate TRPA1 *via* covalent modification of cysteine moieties within the cytoplasmic N terminus of the channel (43), whereas activation of TRPV1 has been reported *via* an interaction with the capsaicin binding site (44). The EC_50_ values for the activation of TRPA1 by AITC vary among reports using TRPA1 overexpressing cells from as little as 0.6 μM (45), to 1.47 μM (46) and 3–34 μM (47). Thus, we next determined the relevance of extracellular calcium influx for the observations made with AITC. Extra cellular calcium content was reduced by addition of the chelating agent EDTA at 0.5 mM before AITC exposure (**Figure 2B**). Our data demonstrate that EDTA treatment did not impact calcium influx triggered by 1 µM AITC (**Figure 2B**). However, at 100 µM AITC, a significant reduction of 67% in calcium flux could be seen upon EDTA treatment indicating a relevance of extracellular calcium at this high AITC concentration. To investigate this further, calcium flux assays were performed using capsazepine as TRPV1 and A-967079 as TRPA1 antagonist. The inhibitors were then used at their reported half maximal inhibitory concentration (IC_50_) and 10 or 100-fold higher. A reduction of AITC mediated calcium release by capsazepine could only been detected by pretreatment with a high concentration of 10 µM before stimulation with 100 µM AITC. Calcium release induced by 1 µM AITC was not effected (**Figure 2C**). Similar results were derived for TRPA1 inhibition. Using the IC_50_ concentration of 67 nM, that has been reported in calcium assays by (48), no reduction of calcium release upon stimulation with 1 µM AITC was detected. Only much higher concentrations of A-967079 blocked the calcium release. A partly reduction of 35% was observed after pretreatment with 10 µM TRPA1 inhibitor in cells activated by 100 µM AITC (**Figure 2D**). These results indicate to a specific TAS2R38 activation by AITC at low concentrations.

Phosphoinositide-specific phospholipase C is a key enzyme in the regulation of IP_3_-mediated calcium release. To clarify the role of PI-PLCβ in AITC-mediated calcium regulation, the PI-PLCβ inhibitor U73122 was used. As given in **Figure 2E**, cell pretreatment with U73122 could almost completely block the AITC induced calcium release.

### AITC Stimulates NO Production andExerts Bactericidal Activity TAS2R38 Genotype Dependent

**Figure 3:**
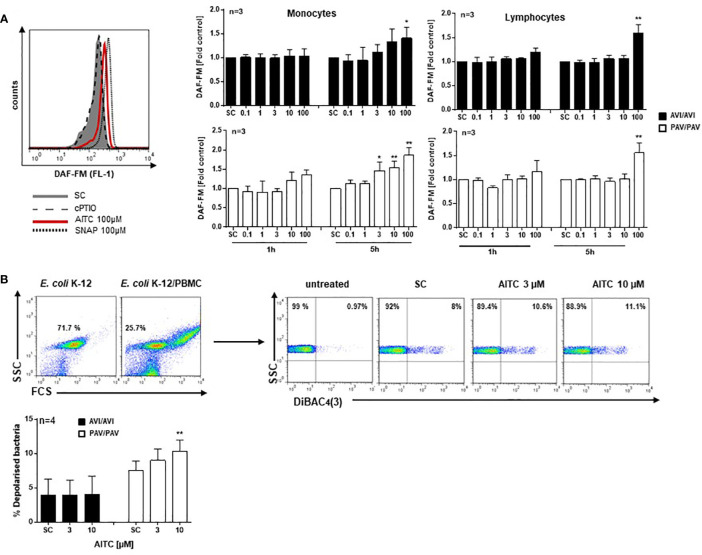
AITC stimulates NO production and exerts bactericidal activity in dependence of the TAS2R38 haplotype. PBMC were isolated from AVI/AVI or PAV/PAV genotype subjects and **(A)** exposed to 0.01% DMSO (SC), AITC, the NO-donor SNAP (positive control), or the NO scavenger cPTIO for the indicated time points. Then, 0.5 µM DAF-FM diacetate was added for 30 min. Fluorescence signals (ex 488 nm, em 530± 30 nm) were monitored using a FACSCalibur™. The median fluorescence intensity (MFI) of each sample was calculated using FlowJo software (Ashland, Oregon, USA). A representative histogram of one PAV/PAV subject is shown on the top, **(B)** incubated with *E. coli* K-12 at MOI 0.5 and exposed to 0.01% DMSO (SC) or AITC for 120 min at 37°C. Cell membrane potential depolarization was determined by staining bacteria with 1 µg/ml DiBAC_4_(3) for 10 min, in the dark at room temperature. Representative scattergrams of *E. coli* K-12 and PBMC treated with *E. coli* K-12 are given. An FSC/SSC plot was first made and *E. coli* K-12 cells gated. The population was copied to a SSC/DiBAC_4_(3)-scatterplot identifying the percentage of depolarized bacteria. Bars are mean value + SD, *p < 0.05, **p < 0.01. Significance of difference was calculated relative to the respective control by one-way ANOVA.

Nitric oxide (NO) is a key feature of immune cells and plays an integral role in defending against pathogens. NO is synthesized by the enzyme NO synthase (NOS), cNOS is constitutively present in the cell and calcium dependent but iNOS is calcium independent and expressed only after stimulus (49). We thus next evaluated the potential of AITC to trigger NO production in response to low-level intracellular calcium changes that are known to stimulate calmodulin-dependent NOS activation (49) using the DAF-FM probe. There was an insignificant increase in NO in the monocyte cell population by AITC (≥ 10 µM) after 1h in cells from PAV/PAV, not AVI/AVI subjects (**Figure 3A**). After 5h exposure to AITC, a genotype-dependent NO production became evident (PAV/PAV: 46% increase at 3 µM; AVI/AVI: 41% increase at 100 µM). In contrast, there was no differential regulation in NO production in the lymphocyte population; NO production could only be detected after 5h exposure to 100 µM AITC.

Human PBMC were then co-incubated with *E. coli* K12 bacteria at multiplicity of infection (MOI) 0.5 and membrane potential changes quantified using DiBAC_4_(3) staining (**Figure 3B**). While no effect could be seen in AITC treated AVI/AVI cells, an increased bactericidal activity of AITC became evident in PAV/PAV cells.

### AITC Modulates the MAPK and PI3K/AKT Signaling Pathway TAS2R38 Genotype Dependent

**Figure 4:**
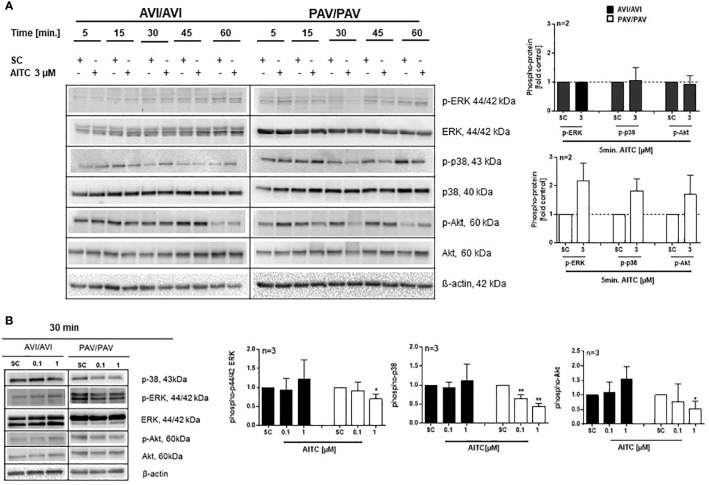
AITC mediates MAPK down-stream signaling in dependence of the TAS2R38 haplotype. **(A**, **B)** PBMC were treated with test compounds for the indicated time points and pellets were collected for protein expression analysis. Representative immunoblots are given. Densitometric analysis was done to quantify the immunoblots using Quantity One software (Bio-Rad, Munich, Germany) and normalized against β-actin, which was used as loading control. The graphs show phosphorylated ERK1/2, p-38 or Akt, protein levels relative to control as quantified by pixel densitometry. Bars are mean + SD; *p < 0.05, **p < 0.01 (n ≥ 2). Significance of difference was calculated relative to the corresponding solvent control determined by one-way ANOVA (SC, 0.01% DMSO).

The mitogen-activated protein kinases (MAPKs) pathway is one of the most widely studied signaling pathways which is essential for processes that are central to inflammatory responses by immune cells, e. g. MAPK signaling is involved in cellular stress response, proliferation and differentiation (50). The phosphatidylinositol 3-kinase (PI3K)/AKT pathway also regulates cell metabolism, growth or survival; AKT is here a primary mediator of the PI3K signaling cascade. In PBMC, a quick activation (5 min. AITC exposure), followed by dephosphorylation of p-ERK1/2 and p-p38 was evident in the PAV/PAV group upon AITC treatment, in contrast to cells from AVI/AVI subjects (**Figure 4**) as compared to control cells. The same activation/inactivation pattern could be seen for p-Akt upon AITC treatment in dependence of the TAS2R38 genotype (**Figure 4**).

### AITC Inhibits TNF-Alpha Secretion of Activated PBMC in a TAS2R38 Genotype Dependent Manner

**Figure 5:**
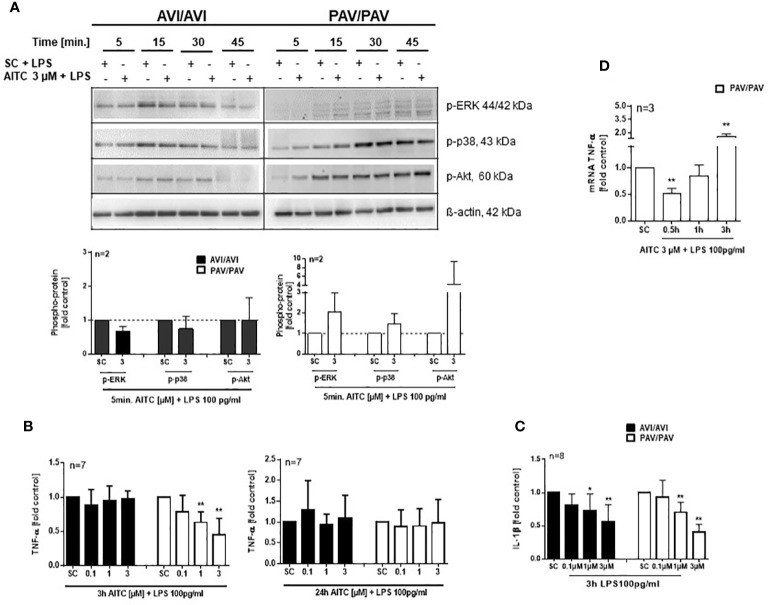
In bacterial LPS stimulated PBMC, AITC triggers MAPK activation and TNF-α inhibition in dependence of the TAS2R38 genotype. PBMC were exposed to AITC or 0.01% DMSO (SC) together with 100 pg/mL LPS. **(A)** Representative immunoblots are given. Densitometric analysis was done to quantify the immunoblots using Quantity One software (Bio-Rad, Munich, Germany) and normalized against β-actin, which was used as loading control. **(B**, **C)** Cytokine secretion at 3 and 24 h was analyzed using ELISA kits. **(D)** Time kinetic of TNF-α mRNA expression. Results are means + SD and given as fold of the solvent control. Results were calculated relative to the corresponding solvent control (SC, 0.01% DMSO). Significance of difference was determined compared to the respective SC by one-way ANOVA, *p < 0.05, **p < 0.01.

**Figure 6:**
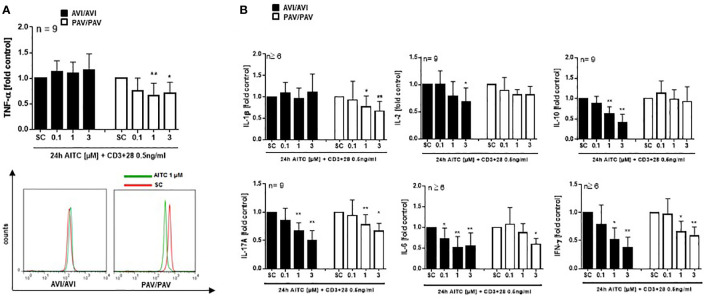
In bacterial LPS stimulated PBMC, AITC triggers MAPK activation as well as TNF-α release in dependence of the TAS2R38 genotype. PBMC were exposed to AITC or 0.01% DMSO (SC) together with 0.5 ng/mL CD3/CD28 MAbs for 24h **(A**, **B)**. Cytokine secretion was analyzed using the human MACSplex cytokine 12-kit or ELISA kits. Results are mean values + SD and given as fold of the solvent control. Significance of difference was determined compared to the respective SC as determined by one-way ANOVA, *p < 0.05, **p < 0.01.

Likewise, an upregulation of p-ERK, p-p38 and p-Akt phosphorylation was evident after 5 min. treatment with 100 pg/mL LPS and AITC in the PAV/PAV subjects (**Figure 5A**). We then addressed the question of whether immune responses vary between TAS2R38 genotype variants in terms of cytokine release. First, we investigated whether cell signaling triggered by mere AITC exposure could mediate cytokine release in the absence of any further inflammatory stimulus such as LPS. However, no change in TNF-α release could be detected upon AITC exposure, then (data not shown). In 3 h LPS activated PBMC, AITC substantially suppressed TNF-α secretion (peak inhibition of 54% at 3 µM) and this was dependent on TAS2R38 receptor functionality (**Figure 5B**). Prolonged incubation with AITC (24 h) abolished the effect seen in the PAV/PAV group at all tested concentrations, suggesting the involvement of TAS2R38 in an early innate immune response. Release of the pro-inflammatory cytokine IL-1β, on the other hand, was suppressed in both AVI/AVI and PAV/PAV groups by AITC (**Figure 5C**). qRT-PCR analysis revealed a strong (50%) but transient inhibition of TNF-alpha mRNA expression in LPS stimulated PBMC from PAV/PAV subjects by AITC (**Figure 5D**). Similar, but less strong effects were seen in CD3/CD28 mAbs stimulated cells derived from PAV/PAV haplotype subjects upon exposure to AITC. No effect on TNF-α was observed in the AVI/AVI group after treatment with AITC (**Figure 6A**). While a differential inhibition of IL-1β was seen in PAV/PAV vs. AVI/AVI cells, a modulation of other pro-inflammatory (IL-2, IL-6 and IL17-A) and also anti-inflammatory (IL-10) cytokines was not clearly genotype dependent (**Figure 6B**).

### AITC Plasma Levels in Human Volunteers After Food Consumption

The pharmacokinetics of oral AITC dosing has been shown so far only in rats (51). Then the peak plasma level was reached within 0.5 h. Thus, we finally investigated AITC plasma levels in human volunteers after single consumption of a normal portion (10 g) mustard preparation containing 24.75 ± 1.76 µmol/g preformed AITC as determined by GC-MS analysis. After 0.5 h, consumption resulted in a blood plasma level of 836.7 ± 67.7 nM, after 1 h of 1001.0 ± 228.3 nM AITC-NAC metabolite (**Table 1**). No other AITC metabolites (AITC-GSH or AITC-CG) could be detected at this time (data not shown).

**Table 1:** Quantification of AITC plasma levels [nmol/L] after single oral intake of a conventional mustard preparation, as determined using LC-ESI-MS/MS.

		Plasma sampling time	
		30 min.	60 min.
Volunteer A (1)	genotype: PAV/AVI	760.5	
Volunteer A (2)		827.2	
Volunteer B (1)	genotype: AVI/PVA	967.6	
Volunteer B (2)		904.9	
Volunteer C (1)	genotype: PAV/PAV	798.5	851.7
Volunteer C (2)		793.1	795.8
Volunteer D (1)	genotype: PAV/PAV	838.1	1057.6
Volunteer D (2)		804.0	1298.9

Plasma was sampled either 30 min. (N=4) or 60 min. (N=2) after intake. Each sample was measured in duplicate.

## Discussion

Food and nutrition are closely linked with health; food may be the cause and also a cure of illness. However, it is well documented that people respond differently to plant components and food–gene interactions may explain why some individuals respond more favorably to food interventions than others. Inter-individual variation in drug response among patients is also well known and poses a serious problem in medicine (52, 53). Here, a better understanding of how diet affects health could be achieved by providing scientific evidence of differential responses to bioactive phytochemicals dependent on individual genetic variations. This could present a decisive step towards individualizing or personalizing food for reaching better disease prevention and treatment strategies.

A single-nucleotide polymorphism in the TAS2R38 receptor gene has decisive influence on individual bitter taste sensitivity and beyond this, increasing evidence indicates a relevance of the TAS2R38 receptor in innate immune defense against microbial attack (8, 26, 27, 54). Our data on the natural TAS2R38 receptor agonist AITC support this hypothesis and further indicate an additional, albeit less strong, role in the adaptive immune response.

The G protein subunit couples activated TAS2Rs to phospholipase C activity, inositol trisphosphate, and this releases calcium from its internal stores (55). Calcium in turn is a second messenger that affects many cell signaling processes. Using the PLCβ-2 inhibitor U-73122, we could confirm that AITC-triggered calcium mobilization predominantly took place through the AITC-TAS2R38-Gαβγ-PLC pathway in PBMC. Further, our findings indicate that the cation channels TRPV1 and TRPA1, which are known to be also a target of AITC (46, 56, 57), do not account for the observations at diet-related concentrations, but only at supra physiological ones. In some cases, wide variations in TAS2R38 dependent calcium release were observed.

This may relate to individual expression levels of TAS2R38, which have been reported previously (58). Moreover, the bitterness ratings of human volunteers for 6-n-propylthiourea (PROP), a prototypical synthetic TAS2R38 agonist, were correlated with the TAS2R38-PAV mRNA levels. Even though these experiments were done in individuals heterozygous for TAS2R38-taster and non-taster alleles, this suggests that TAS2R38 function is directly linked to TAS2R38-PAV expression levels (58). For the observed discrepancy between AITC threshold concentration in monocytes (10 nM) and published *in vitro* data of the heterologous expressed TAS2R38-PAV (10 µM) (18) we have no immediate explanation. Different experimental settings, such as different cell types may partially account for this. Also, we assessed calcium-dependent changes of cellular fluorescence after prolonged exposure to AITC. This is different to the *in vitro* heterologous experiments where, due to the transient nature of calcium signals in HEK cells, peak fluorescence is reached within seconds after substance application, although the stimulus is usually still present. Hence, the rather long accumulation of AITC-stimulated calcium ions might have shifted the detection limit towards lower concentrations.

The MAPK ERK1/2 is activated preferentially by mitogenic factors, differentiation stimuli and cytokines, while p38 MAPKs respond to conditions of cellular stress (59). MAPK activities are thereby subject to negative feedback control. The dephosphorylation of MAPKs plays here a key role in determining the magnitude and duration of kinase activation and hence the physiological outcome of signaling. For ERK1/2, a calcium/calmodulin/calcineurin-dependent protein inactivation has been described before (60). In the present study, a quick activation, followed by dephosphorylation of ERK1/2 and p38 at their Thy/Thr phosphorylation sites and also Akt at Ser473 could be seen upon AITC exposure but only in cells from PAV/PAV individuals which further supports the idea of a distinct regulation of key signal transduction pathways dependent on TAS2R38 activation.

NO is a short-lived free radical, recognized as a highly cytotoxic and ubiquitous biomessenger molecule. NOS is a calmodulin binding protein and thus regulates the NO pathway (61). In sinonasal cells, NO production was found to be stimulated by the TAS2R38 agonist PTC in a TAS2R38 genotype dependent manner (28). Its inducible form (iNOS) is absent from resting immune cells, and is expressed in response to stimuli, e. g. microbial LPS. Macrophages are here considered the prototypic iNOS expressing cells (62–64). This concurs with our observation that AITC-mediated NO production was mainly seen in the monocyte subpopulation of PBMC. Then, an earlier and more intense response of PAV/PAV cells to AITC as compared to AVI/AVI was seen. A higher NO production was also detected in human PAV/PAV epithelial cells > PAV/AVI > AVI/AVI upon calcium flux triggered by TAS2R38 sensitive quorum sensing molecules or PTC. This activation then ultimately resulted in increased bactericidal activity (26, 28). The authors thus proposed a specific role of TAS2R38-induced NO production, which could facilitate the bactericidal action for PAV/PAV genotypes (26, 28). In our experiments such increase in bactericidal action could be confirmed for PAV/PAV immune cells against *E. coli* K12 bacteria.

The immune response to pathogens involves the rapid activation of pro-inflammatory and anti-inflammatory mechanisms that serve to initiate host defense against microbial invasion and in parallel maintain or restore tissue homeostasis (65). TNF-α is, besides a number of other inflammatory cytokines, pleiotropic and critical in regulating cellular stress responses, metabolism, and even food intake (66–68). Results in TNF-deficient mice suggest that the cytokine itself is involved in bitter taste reception regulation (69). Treatment with denatonium, which activates multiple TAS2Rs, triggered a marginal change in cytokine release from sinonasal epithelial cells (70). A marked reduction of multiple cytokine production, including TNF-α, has been shown in LPS stimulated leukocytes exposed to denatonium or other T2R agonists (71).

Compound concentrations that prove to be active *in vitro* may not be reached in the human organism, e. g. due to a reduced bioavailability. So far, no data of human AITC plasma levels had been reported upon food consumption or pharmaceutical intake. Here, we could now show that single intake of an AITC containing food product resulted in AITC plasma levels of around one µM. This indicates to a sufficient systemic bioavailability after normal food intake to trigger TAS2R38-dependent immune cell responses. Whether the *in vitro* findings can be translated into a clinically relevant setting will now have to be investigated in a well-designed randomized controlled trial.

## Conclusion

So far, most of the available scientific evidence in support of precision nutrition is based on observational studies. Much more research will be required before personalized nutrition can deliver the expected benefits. The present study could now provide first biological evidence of differential immune responses to the food-born compound AITC dependent on a genotypic characteristic. Across the globe, plants from the order *Brassicales* are very popular, and considered as “healthy” despite them tasting bitter for many people. Besides food item, they are used in traditional medicine (e. g. “mustard plasters” against bronchitis) and pharmaceutical remedies against bacterial infection and inflammation. Maybe in future, individuals could be identified based on their genetically determined individual taste perception that profit more from an AITC-containing food intervention or be more responsive to such a medical treatment than others. This could then guide timely alternative (therapeutic) interventions in people who inherit nonfunctional TAS2R38 alleles. However, until we arrive at this point clearly more research is needed. Interestingly, in a study by Meyerhof et al. (18) AITC as well as the structurally related phenylethyl ITC activated the TAS2R38-PAV receptor. In contrast, another ITC, L-sulforaphane, did not. So there seem to be some other, yet unknown factors determining the activation capacity and one may not generalize our results on all compounds from this class.

## Data Availability Statement

The original contributions presented in the study are included in the article/supplementary material. Further inquiries can be directed to the corresponding author.

## Ethics Statement

The studies involving human participants were reviewed and approved by the Ethics Committee of the University of Freiburg. The patients/participants provided their written informed consent to participate in this study.

## Author Contributions

EL designed the study and experiments. HT, RS, CH, JS, MK, and FH designed and carried out the experiments. HT, RS, and EL prepared the graphs and analyzed the data. FH analyzed AITC in mustard samples. EL, SR and MS provided study materials and reagents. EL drafted the first version of the manuscript. EL, HT, RS, and MB wrote the paper. All authors contributed to the article and approved the submitted version.

## Funding

The article processing charge was partly funded by the German Research Foundation (DFG) and the University of Freiburg in the funding program Open Access Publishing. FH is funded by the Leibniz-Association (Leibniz-Junior Research Group OPTIGLUP; J16/2017).

## Conflict of Interest

A part of the study was financed by a grant from Repha gmbh, Langenhagen, Germany. Repha GmbH was not involved in the study design, interpretation of the results or writing of the manuscript.

The authors declare that the research was conducted in the absence of any commercial or financial relationships that could be constructed as a potential conflict of interest.
